# Biosimilars: Beginning a Conversation

**DOI:** 10.6004/jadpro.2016.7.3.1

**Published:** 2016-04-01

**Authors:** Christopher J. Campen, Kelley D. Mayden, Ali McBride, Michael Swit

**Affiliations:** 1 Greenville Health System, Greenville, South Carolina;; 2 Wellmont Cancer Institute, Bristol, Virginia;; 3 University of Arizona Cancer Center, Tucson;; 4 Illumina, Inc.

In 2010, the Biologics Price Competition and Innovation (BPCI) Act was passed under the Patient Protection and Affordable Care Act (FDA, 2010). It created an abbreviated pathway for the approval of biologic products demonstrated to be clinically similar (or biosimilar) to or interchangeable with an approved reference product.

A diverse panel of experts convened at JADPRO Live at APSHO to discuss key requirements for interchangeability and potential roadblocks to US Food and Drug Administration (FDA) pathways for the approval of biosimilars.

Panel moderator Christopher J. Campen, PharmD, BCPS, BCOP, clinical pharmacist, Greenville Health System, Greenville, South Carolina, kicked off the discussion by asking why the United States lags behind Europe in bringing biosimilars to market. Kelley D. Mayden, MSN, FNP, AOCNP®, nurse practitioner, Wellmont Cancer Institute, Bristol, Virginia, cited incomplete draft guidance from the FDA. As a result, she said, the pharmaceutical industry "didn’t really understand exactly what was expected," and further had to evaluate whether the biosimilar market was lucrative enough to invest in.

The FDA took the stance that it didn’t have the unilateral authority to create a biosimilar pathway, so it was dependent on legislation, "and that took a long time because that’s the way Washington, DC, works," commented Michael Swit, Esq, Senior Director, Legal, Regulatory at Illumina, Inc., San Diego, California. "The FDA never wants to issue a regulation if they don’t have to because it becomes binding on them. So they issue guidance documents, and that takes a long time, in part because you don’t have user fees associated with the whole biologic process."

Regulatory and pricing issues initially held back the biosimilar market in the United States, but the pathway to approval guidance from the FDA has improved over the past 5 years, according to Ali McBride, PharmD, BCPS, BCOP, Clinical Coordinator, University of Arizona Cancer Center, Tucson.

## INTERCHANGEABILITY VS. EXTRAPOLATION

The panel moved to key requirements for a biosimilar agent to be deemed interchangeable. Interchangeability is defined as no difference in outcome when switching between products and is much like the practice of therapeutic interchange, said Dr. McBride. In contrast, extrapolation is extending biosimilar approval to all FDA-labeled indications.

Mr. Swit stated that the BPCI Act requires that a biosimilar demonstrate clinical equivalence in any given patient. This requirement compels biosimilar manufacturers to conduct expensive clinical trials to demonstrate an equivalent clinical effect to an innovator product, with no major difference in side effects for all indications that a biosimilar manufacturer wishes to pursue. The end result may be that biosimilar manufacturers will attempt to extrapolate interchangeability for all indications when approaching Pharmacy & Therapeutics committees for formulary inclusion, he said.

Dr. McBride argued that doing clinical trials for every single indication when a biosimilar has already demonstrated interchangeability for one indication would substantially increase the cost of the biosimilar, removing the main incentive for its use.

Much of the guidance with respect to biosimilars will borrow heavily from Europe, which "does a fantastic job of looking at all the biosimilars and their outcomes," he said. "One of the key takeaway points is that we are using a lot of the content from the European experience to apply our principal foundations for biosimilar interchangeability."

"I have been saying for quite some time that this is not your father’s generic drug model," said Mr. Swit. "This is a brand in play. The biosimilars are going to be marketed like branded products. Personally, I would love to see widespread interchangeability and adoption of biosimilars. I hope that I’m wrong about the ’any given patient’ language of the statute."

He added that off-label use of a biosimilar would be at the discretion of the prescribing physician, as is off-label use of any product currently, and that recent court cases have established a First Amendment right for pharmaceutical manufacturers to market their products off-label. "You’re going to see a lot of outcomes data coming in from the biosimilar market and pushing for the use off-label," he said.

Ms. Mayden wondered about the implications of off-label use of biosimilars as far as reimbursement from third-party payers. Dr. McBride answered that, much as is the case with all therapies, "if you don’t have the data, then you may not be able to prescribe that drug therapy." He continued, "I do think we are waiting on what payers…are going to be stating on switching." Payers are already inducing cost-cutting switches, such as tbo-filgrastim (Granix) for filgrastim (Neupogen), he said.

Widespread off-label adoption of biosimilars may hinge on National Comprehensive Cancer Network (NCCN) guidance, believes Dr. McBride. Whether or not extrapolation into NCCN guidelines occurs is "the key question we will be holding on to over the next few years, as more and more biosimilars come on to the marketplace," he said.

Ms. Mayden asked about the level of confidence needed before P&T committees believe that they can safely interchange biosimilars. Dr. McBride said that although surrogate endpoints may suffice for biologic treatments for anemia, "when it comes to therapeutic efficacy, like rituximab (Rituxan) and overall survival…I think we have a lot more cynicism in evaluating that content and making sure that we feel appropriate with the data endpoints." In this case, data from more than one clinical trial may be needed to instill confidence in interchangeability.

## ENSURING PHARMACOVIGILANCE

Once the biosimilars enter the health-care system, "pharmacovigilance" will be crucial, said Dr. McBride. Patients and nursing staff alike will have to be included in the education.

FDA guidance for naming biosimilars was issued in August 2015, which should help in transitions of care, as payers may dictate the switching of products, he noted. In its guidance, the FDA encouraged the addition of suffixes to distinguish between biologic products irrespective of their licensure pathway. Clearly, different names for clinically similar products would enhance pharmacovigilance, said Mr. Swit.

Use of the National Drug Code (NDC), which assigns unique codes to individual products, may also help in distinguishing between biosimilars, said Dr. McBride, although the FDA stated in its draft guidance that "use of distinct proprietary names or NDC numbers is insufficient to address concerns regarding pharmacovigilance." He also envisions that Flatiron Health, a cloud-based health-care technology company, will eventually be used to pull information from the electronic health record to simplify pharmacovigilance and perhaps assist in tagging outcomes content specific to biosimilars.

"The pharmacovigilance systems tend to be the same whether you are a small molecule that has been approved under the Food, Drug, and Cosmetic Act or a biologic approved under the Public Health Service Act," said Mr. Swit. "Why this is suddenly, magically different, I don’t fully understand. One could argue that this whole need for a suffix is a fiction created by the brand name companies to try to insulate them from competition."

Said Dr. Campen, "at the same time, we know from the European examples that there were issues in some of the earlier studies with immunogenicity." Examination of immunogenicity will become part of the pharmacovigilance pathway, said Dr. McBride, and will be incorporated into assessment of similarity, because antibodies binding against a biologic agent will most likely result in inactivation and decreased efficacy, differences in side-effect profile, and changes in the rate of infusion reactions.
